# Low antithrombin levels are associated with low risk of cardiovascular death but are a risk factor for cancer mortality

**DOI:** 10.1371/journal.pone.0271663

**Published:** 2022-09-19

**Authors:** Licia Iacoviello, Romy de Laat-Kremers, Simona Costanzo, Qiuting Yan, Augusto Di Castelnuovo, Lisa van der Vorm, Amalia De Curtis, Marisa Ninivaggi, Chiara Cerletti, Maria Benedetta Donati, Bas de Laat, on behalf of the Moli-sani Investigators

**Affiliations:** 1 Department of Epidemiology and Prevention, IRCCS Neuromed, Pozzilli, Italy; 2 Research Center in Epidemiology and Preventive Medicine (EPIMED), Department of Medicine and Surgery, University of Insubria, Varese, Italy; 3 Department of Data Analysis and Artificial Intelligence, Synapse Research Institute, Maastricht, The Netherlands; 4 Department of Functional Coagulation, Synapse Research Institute, Maastricht, The Netherlands; 5 Department of Biochemistry, CARIM, Maastricht University, Maastricht, The Netherlands; 6 Mediterranea Cardiocentro, Napoli, Italy; Karolinska Institutet, SWEDEN

## Abstract

**Background:**

Thrombosis is common in subjects suffering from cardiovascular diseases (CVD) and cancer. Hypercoagulation plays a pivotal role in the pathophysiology of thrombosis. Therefore, the inactivation of thrombin, the key enzyme in coagulation, is tightly regulated via antithrombin (AT). AT deficiency is related to thrombosis and cardiovascular death. In this study we investigated the association between AT levels and mortality, in particularly cardiovascular-related and cancer-related death in the general population.

**Methods:**

We studied the association of AT levels and mortality in a prospective cohort sampled from the general Italian population (n = 19,676). AT levels were measured in the baseline samples, and mortality was recorded during a median follow-up period of 8.2 years. Cox regression was performed to investigate the association of all-cause, CVD-related and cancer-related mortality with variations in AT levels.

**Results:**

In total, 989 subjects died during follow-up, of which 373 subjects of CVD and 353 of cancer-related causes. Cox analysis revealed that, after adjustment for age, sex, current smoking, BMI, diabetes, hypertension, hypercholesterolemia, history of cardiovascular disease, history of cancer, vitamin K antagonists, antiplatelet medication, heparin and oral contraceptives AT levels were not associated with all-cause mortality (HR_Q1vsQ5_: 0.92, 95% CI:0.74–1.15). Interestingly, the risk of CVD-related mortality was reduced in subjects with low AT levels compared to subjects with higher AT levels, after adjustment for age and sex and other confounders did not change the association (HR_Q1vsQ5_: 0.64, 95% CI:0.44–0.91). Moreover, low AT levels were associated with increased cancer mortality in a fully adjusted model (HR_Q1vsQ2-5_: 1.26, 95% CI:0.88–1.81).

**Conclusions:**

Low AT levels are associated to a lower risk of fatal cardiovascular events in the general population, regardless of age, sex and medication use. In contrast, low AT levels are associated with lower cancer survival. For the first time we show that AT levels lower than the normal range in the general population, even before the development or diagnosis of cancer, are associated with an elevated risk of cancer death.

## Introduction

The leading causes of death worldwide are ischemic heart disease and stroke, which affect approximately 15.2 million people every year according to the World Health Organization. Heart disease and stroke are caused by vascular occlusions mostly deriving by thrombus formation and thus by changes in the coagulation system and in vascular wall functions The key enzyme in hemostasis is thrombin, the enzyme that cleaves fibrinogen into fibrin and forms a fibrin clot to prevent blood loss after vessel injury. On the contrary, pathological clot formation that occurs within a blood vessel causes infarction of tissue, for example in the heart (myocardial infarction) or in the brain (ischemic stroke). Patients suffering from cancer are in a hypercoagulable state and particularly at risk for thrombosis [1]. The risk of developing venous thromboembolism is 4-7-fold higher in cancer patients compared to the general population and occurs in up to 20% of all cancer patients [2, 3]. Additionally, arterial thrombosis is estimated to occur in 2–5% of cancer patients [4]. The higher incidence of thrombotic events in cancer patients is related to general risk factors, such as age, immobility and obesity [1], cancer-related factors (e.g. tumor tissue factor expression and Cancer Procoagulant [5, 6]) and cancer treatment specific risk factors (e.g. chemotherapy [7, 8]).

In general, a low thrombin generation capacity is associated with bleeding and a high thrombin generation with thrombosis. Subsequently, the production and inactivation of thrombin is tightly regulated in coagulation to prevent thrombosis and bleeding, while maintaining hemostatic balance. The main natural anticoagulant present in plasma is the thrombin inhibitor antithrombin (AT). AT is a serine protease inhibitor that, besides thrombin, also inhibits coagulation factors Xa, IXa, XIa, kallikrein and plasmin [9]. Inherited deficiencies in AT cause thrombotic complications. AT deficiency is caused by an inherited gene defect or can be acquired later in life, such as impaired AT production in liver cirrhosis patients, or in certain kidney diseases. Inherited AT deficiency occurs in 0.2–0.02% of the general population [10]. Homozygotic AT deficiency is not compatible with life and heterozygotic AT deficiency is associated with arterial clots, such as strokes and myocardial infarction [10–12]. AT deficiency leads to thrombotic complications, such as deep venous thrombosis and pulmonary embolism [10, 13]. Approximately half of the individuals with AT deficiency will develop venous thrombosis during their life [10, 13].

Since AT plays a dominant role in the inhibition of coagulation, it is an interesting therapeutical target. Heparins accelerate the inhibitory actions of AT on thrombin and FXa and are widely used as anticoagulant. On the contrary, AT concentrates are administered to replace AT in e.g. sepsis and cancer patients, to prevent disseminated intravascular coagulation and improve the patients outcome in general [14]. A novel treatment in hemophilia is the targeting of AT by a silencing miRNA in hemophilia [15], which reduces AT levels in an attempt to lower the anticoagulant response and to match the lower pro-coagulant response in hemophilia patients.

Although heterozygotic AT deficiency is a well-known risk factor for thrombosis, it has never been investigated whether subjects with AT levels at the low and/or high borders of the normal range have an elevated risk of cardiovascular or cancer disease-related mortality. It is unclear whether the hemostatic outcome in subjects within the defined boundaries of the normal range for AT are in fact comparable for the whole range of AT, or whether AT levels should be considered on sliding scale for clinical outcomes even in the normal range. In this prospective study we set out to determine in the general population the association of AT with mortality, both specifically CVD-related and cancer-related mortality.

## Materials and methods

### Study population

The subjects of the Moli-sani cohort were randomly recruited in the Molise region (Italy) from city hall registries, as previously described [16, 17]. The Moli-sani study complies with the Declaration of Helsinki and was approved by the ethics committee of the Catholic University of Rome, Italy. Between March 2005 and April 2010, 24,325 subjects were enrolled. All participants provided written informed consent.

### Sample collection

Venous blood samples were obtained by venipuncture between 07:00 am and 09:00 am from participants who had fasted overnight and had refrained from smoking for at least 6 hours [16]. Citrated plasma samples for this study were initially stored in straws containing the sample code and barcode in liquid nitrogen in a dedicated biobank (http://www.neuromed.it/biobanking-centre/). They were express-shipped in 3 batches on dry ice to Synapse Research Institute, Maastricht, the Netherlands, where they were immediately stored at -80°C. Levels of labile coagulation factors (FV, FVIII, and FIX) were determined in a subset of 144 samples from the first batch to confirm plasma sample quality. All coagulation factors measured were within reference normal ranges according to the assay instructions for use.

### Measurement of AT levels

Plasma AT levels were measured in 20,521 samples, at the Synapse Research Institute laboratories, Maastricht (the Netherlands), on the STA-R Max (Diagnostica Stago, France) using the STA-Stachrom AT III reagent according to the manufacturer recommendations. The analytical reporting range was 9%-160% and the intra- and inter-assay variation were 2.6% and 4.8%, respectively. The reference range for AT in the general population as reported in literature is 71–130% [18].

### Baseline data collection

The baseline visit included questionnaires about socioeconomic status, physical activity, medical history, dietary habits, risk factors, personal and family medical history; measurements of blood pressure and anthropometry; collection of morning venous blood samples after overnight fasting. Body Mass Index (BMI) was calculated as weight/(height)^2^ (kg/m^2^).

The dataset of the Moli-sani Study provides accurate information on the use (frequency, dose, compliance) of medication for any disease, collected during the recruitment. The questionnaire on drug use was directly linked to the Italian National drug index. Use of vitamin K antagonists, antiplatelet medication, heparin, and oral contraceptives was recorded. History of CVD (including angina, acute myocardial infarction, revascularization procedures, cerebrovascular events and peripheral artery disease) were self-reported and confirmed by medical records. History of cancer was self-reported.

Blood pressure was measured at time of inclusion and blood sampling, by an automatic device (OMRON-HEM-705CP) three times on the non-dominant arm, with the patient lying down for about 5 min, and the average of the last two values was taken as the BP. Hypertension was defined as systolic BP (SBP) ≥ 140 mmHg or diastolic BP (DBP) ≥ 90 mmHg or based on current treatment with antihypertensive drugs. Pre-hypertension was defined as SBP of 130–139 mmHg or DBP of 85–89 mmHg.

Serum lipids (HDL-cholesterol, triglycerides) and blood glucose were assayed by enzymatic reaction methods using an automatic analyzer (ILab 350, Instrumentation Laboratory [IL], Milan, Italy). Hypercholesterolemia was defined as serum total cholesterol ≥ 240 mg/dL or current treatment with Cholesterol Medications. Pre-hypercholesterolemia was defined as serum total cholesterol of 200–239 mg/dL. Diabetes was defined as serum glucose ≥ 126 mg/dL or based on current treatment with Diabetes Medications. Pre-diabetes was defined as serum glucose of 110–125 mg/dL.

### Follow-up data collection

During follow-up (up to 31 December 2015), mortality was recorded as total, cardiovascular disease-related mortality, or cancer-related mortality using the Italian mortality registry (ReNCaM) and validated using Italian death certificates. Deaths were coded according to the International Classification of Diseases-9th Revision (ICD-9). CVD mortality included deaths from diseases of the circulatory system, when the underlying cause of death included ICD-9 codes 390 to 459. Cancer death was considered when the underlying cause of death included ICD-9 codes 140 to 208. Deaths that were neither b CVD nor cancer-related were included in the “other-cause mortality” group. The median follow-up time for mortality was 8.2 years (interquartile range: 7.2 to 9.2 years; 161,662.6 person-years).

Primary incident cases of CHD (unstable angina, myocardial infarction, coronary revascularization and sudden death for unspecified cardiac event) and stroke that occurred in the cohort during follow-up were ascertained by linkage of the study cohort to the hospital discharge files and to the regional ReNCaM registry and death certificates (ISTAT form), by using the International Classification of Diseases, ninth revision (ICD-9). For CHD, ICD 9 codes 410–414 and/or reperfusion procedure (ICD-9 codes 36.0–36.9) and for cerebrovascular disease, ICD9 codes 430–432, 434, 436–438 or procedure codes for carotid revascularization (ICD 9 code 38.12) were considered.

### Data selection

**Fig 1:**
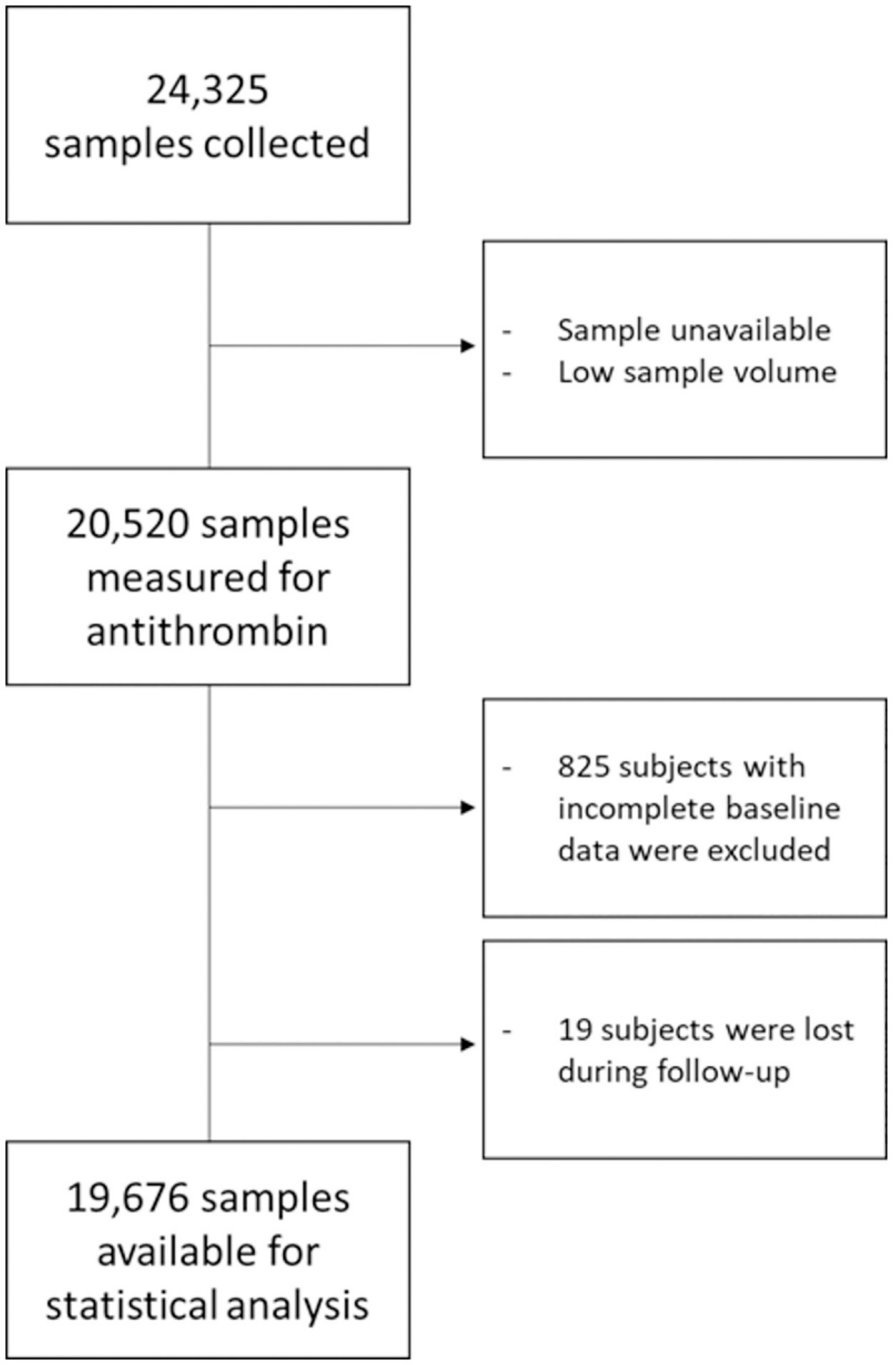
Flow chart of the selection of subjects for statistical analysis.

For the current study, subjects with missing AT data (n = 3805, mainly due to insufficient plasma volumes), incomplete baseline questionnaires (n = 825) and subjects that were lost during follow-up (n = 19) were excluded, leaving 19,676 observations for statistical analysis (Fig 1).

### Statistics

Statistical analysis was performed in SPSS (IBM, Armonk, United States and SAS/STAT software, version 9.4 (SAS Institute Inc., Cary, NC, USA). For analysis, AT levels were split into quintiles. Risk estimates for mortality were expressed as hazard ratios (HR) with 95% confidence intervals (CI), which were calculated using Cox regression models. Three models were analyzed, crude analysis (model 1), sex and age adjusted (model 2) and fully adjusted for current smoking, BMI, diabetes, hypertension, hypercholesterolemia, history of cardiovascular disease, history of cancer, vitamin K antagonists, antiplatelet medication, heparin and oral contraceptives (model 3). Covariates were selected based on correlations existing in the dataset and associations reported in the literature. In a secondary analysis, we estimated mortality risk comparing the lowest quintile up to 94.3% of AT *vs* quintiles 2–5. Adjusted survival curves were constructed for each outcome (all-cause mortality, cardiovascular disease and cancer related mortality) to show event rates during follow up by quintile groups. p-values below 0.05 were considered statistically significant.

## Results

In the Moli-sani cohort, the average AT level was 102.4% ± 10.8%, ranging from 1.42% to 184.84%, 0.72% (N = 142) of values were below 70% (S1 Fig). AT levels were split into quintiles and subjects were grouped in five classes: below 94.3%, 94.3% to 100.5%, 100.5% to 105.4%, 105.4% to 111.1%, and above 111.1% (Table 1). Subjects in the lower quintiles were significantly older and more predominantly men, had a significantly higher BMI and smoked less often. Moreover, hypertension, diabetes, and a history of CVD or cancer were more common in subjects with low AT levels than subjects with higher AT levels. The use of drugs influencing coagulation (vitamin K antagonists and antiplatelet medication) was more prevalent in subjects in the lowest quintiles of AT; in contrast, less women used oral contraceptives.

**Table 1:** Baseline characteristics according to quintiles of AT in the Moli-sani cohort.

	Quintile 1	Quintile 2	Quintile 3	Quintile 4	Quintile 5	*p- value*
	AT < 94.3%	94.3% ≤ AT < 100.5%	100.5% ≤ AT < 105.4%	105.4% ≤ AT < 111.1%	AT ≥ 111.1%	
**N**	3,935	3,936	3,934	3,937	3,934	
**Age (y), mean ±SD**	59.3 ±12.2	56.1 ±11.7	54.1 ±11.5	53.6 ±11.3	53.1 ±10.8	< .0001
**Sex (male), n (%)**	2,545 (64.7)	1,953 (49.6)	1,779 (45.2)	1,630 (41.4)	1,491 (37.9)	< .0001
**BMI (kg/m** ^**2**^ **), mean ±SD**	29.4 ±5.0	28.6 ±4.9	27.7 ±4.7	27.5 ±4.5	26.8 ±4.3	< .0001
**Current smoking, n (%)**	887 (22.5)	904 (23.0)	939 (20.3)	922 (23.4)	964 (24.5)	0.27
**Hypertension status, n (%)**						< .0001
*Pre-Hypertension*	523 (13.6)	569 (14.5)	618 (15.7)	624 (15.9)	673 (17.1)	
*Hypertension*	2,548 (64.8)	2,276 (57.8)	2,017 (51.4)	2,023 (51.4)	2,051 (52.1)	
**Diabetes status, n (%)**						< .0001
*Pre-Diabetes*	514 (13.1)	474 (12.0)	433 (11.0)	416 (10.6)	420 (10.7)	
*Diabetes*	566 (14.4)	376 (9.6)	296 (7.5)	309 (7.9)	261 (6.6)	
**Hypercholesterolemia status, n (%)**						< .0001
*Pre-Hypercholesterolemia*	1,235 (31.4)	1,338 (33.9)	1,314 (33.4)	1,376 (35.0)	1,393 (35.4)	
*Hypercholesterolemia*	984 (25.0)	1,126 (28.6)	1,178 (29.9)	1,268 (32.2)	1,481 (37.7)	
**History of CVD, n (%)**	327 (8.3)	250 (6.4)	161 (4.1)	192 (4.9)	159 (4.0)	< .0001
**History of cancer, n (%)**	148 (3.8)	133 (3.4)	112 (2.9)	119 (3.0)	124 (3.2)	0.18
**Vitamin K antagonists, n (%)**	28 (0.7)	14 (0.4)	7 (0.2)	13 (0.3)	11 (0.3)	0.0016
**Antiplatelet medication, n (%)**	336 (8.5)	261 (6.6)	195 (5.0)	192 (4.9)	173 (4.4)	< .0001
**Heparin use, n (%)**	4 (0.1)	8 (0.2)	6 (0.2)	6 (0.2)	8 (0.2)	0.78
**Oral contraceptives, n (%)** *	163 (11.7)	167 (8.4)	172 (8.0)	152 (6.6)	128 (5.2)	< .0001

* in females

In the Moli-sani cohort, 989 out of 19,676 subjects died (5.03%) during the median follow-up period of 8.2 years. From all deaths that occurred during the follow-up period, 373 deaths were attributed to cardiovascular diseases (37.7%), 353 to cancer (35.7%) and the remainder to other causes. Total mortality rate and CVD-related and cancer-related mortality rates were significantly higher in the lower quintiles of AT.

**Fig 2:**
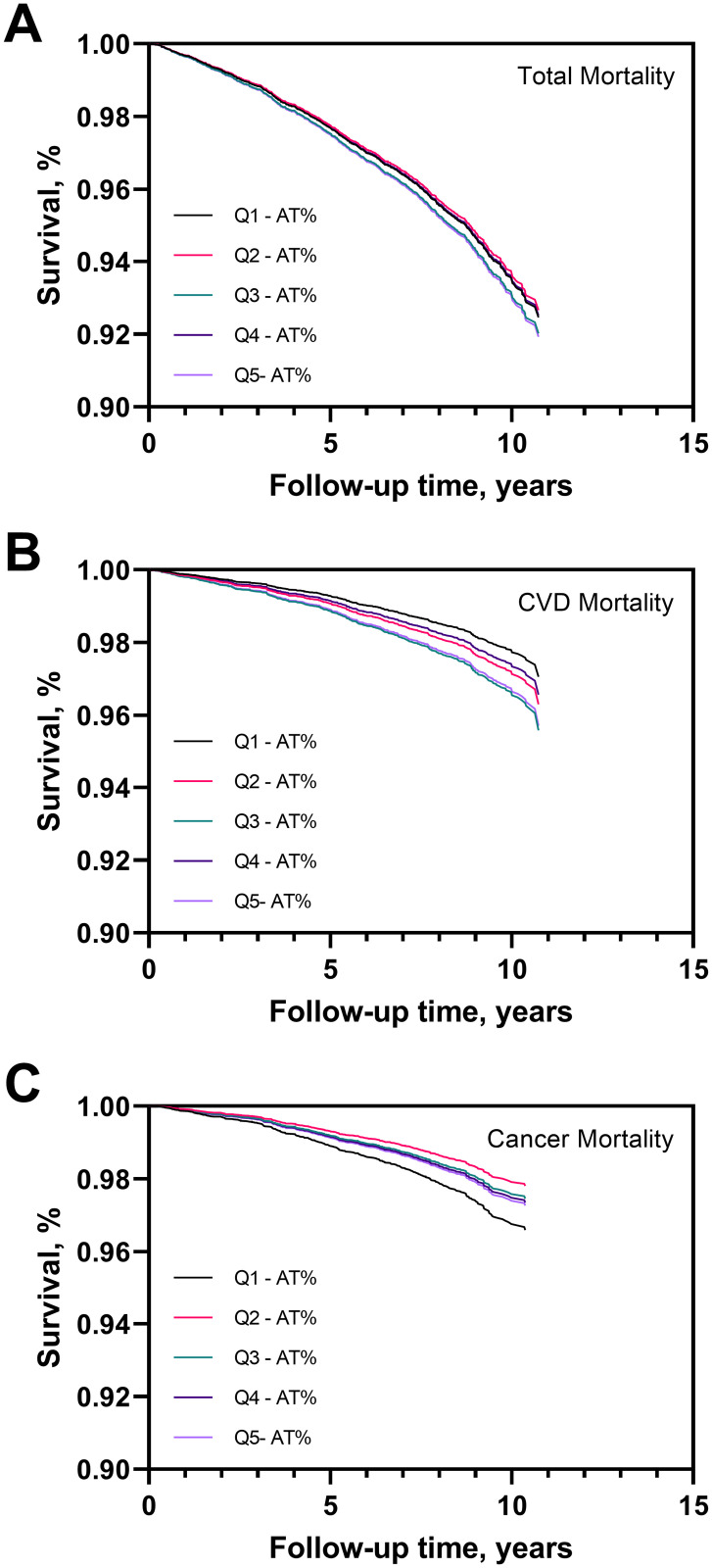
Survival curves according to quintiles of AT in the Moli-sani population (n = 19,676). Survival curve for total mortality (A), cardiovascular disease related mortality (B) and cancer mortality (C) for the quintiles of AT. The curves were adjusted for age, sex, current smoking, BMI, diabetes status, hypertension status, hypercholesterolemia status, history of cardiovascular disease, history of cancer, vitamin K antagonists, antiplatelet medication, heparin use, oral contraceptives.

Fully adjusted survival curves across quintiles of AT levels and mortality showed that cardiovascular disease-related mortality rate was lowest in the lowest quintiles of the distribution, while cancer related mortality was the highest. No significant association between AT categories and all-cause mortality was observed (Fig 2). The association of low AT levels with mortality was investigated in more detail through Cox regression (Table 2). In crude analysis individuals in the lowest quintile of AT (below 94.3%) showed higher total mortality risk as compared to the highest (Q5) (HR_Q1vsQ5_: 2.88, 95% CI: 2.35–3.53; model 1; Table 2). However, after adjustment for age and sex (model 2), the association becomes insignificant and was not modified by further adjustment for current smoking, BMI, diabetes, hypertension, hypercholesterolemia, history of cardiovascular disease, history of cancer, vitamin K antagonists, antiplatelet medication, heparin and oral contraceptives (model 3).

**Table 2:** Incidence and hazard ratios (95% CI) for total, cardiovascular and cancer mortality according to quintiles of AT %, in the Moli-sani population (n = 19,676).

	Quintiles					*p*- for heterogeneity	*p*-value for trend
	Q1	Q2	Q3	Q4	Q5		
Range	AT < 94.3%	94.3% ≤ AT < 100.5%	100.5% ≤ AT < 105.4%	105.4% ≤ AT < 111.1%	AT ≥ 111.1%		
**Total mortality**							
**N**	3935	3936	3934	3937	3934		
**Person Years**	31490.5	32063.9	32478.1	32694.3	32935.8		
**N of events (rate)**	341 (8.7)	210 (5.3)	165 (4.2)	146 (3.7)	127 (3.2)		
**model 1**	2.88 (2.35–3.53)	1.73 (1.38–2.15)	1.33 (1.06–1.68)	1.17 (0.92–1.48)	-1-	< .0001	< .0001
**model 2**	1.04 (0.84–1.29)	0.95 (0.76–1.19)	1.00 (0.79–1.26)	0.97 (0.77–1.24)	-1-	0.89	0.67
**model 3**	0.92 (0.74–1.15)	0.89 (0.71–1.12)	0.99 (0.78–1.25)	0.91 (0.72–1.16)	-1-	0.80	0.50
**CVD Mortality**							
**N**	3931	3931	3934	3932	3934		
**Person Years**	31462.4	32029.6	32478.1	32671.3	32913.1		
**N of events (rate)**	111 (2.8)	87 (2.2)	73 (1.9)	52 (1.3)	50 (1.3)		
**model 1**	2.38 (1.70–3.32)	1.82 (1.28–2.57)	1.49 (1.04–2.14)	1.05 (0.71–1.55)	-1-	< .0001	< .0001
**model 2**	0.74 (0.52–1.04)	0.91 (0.64–1.29)	1.07 (0.74–1.43)	0.84 (0.57–1.24)	-1-	0.16	0.10
**model 3**	0.64 (0.44–0.91)	0.83 (0.58–1.20)	1.04 (0.72–1.49)	0.76 (0.51–1.13)	-1-	0.019	0.021
**Cancer Mortality**							
**N**	3931	3931	3934	3932	3934		
**Person Years**	31462.4	32029.6	32478.1	32671.3	32913.1		
**N of events (rate)**	140 (3.6)	61 (1.6)	53 (1.4)	53 (1.4)	46 (1.2)		
**model 1**	3.26 (2.34–4.55)	1.38 (0.94–2.03)	1.18 (0.79–1.75)	1.17 (0.79–1.73)	-1-	< .0001	< .0001
**model 2**	1.51 (1.06–2.14)	0.89 (0.60–1.32)	0.95 (0.64–1.42)	1.04 (0.70–1.54)	-1-	0.0031	0.016
**model 3**	1.26 (0.88–1.81)	0.79 (0.53–1.17)	0.93 (0.62–1.38)	0.97 (0.65–1.44)	-1-	0.039	0.18

Model **1**: crude; Model **2**: adjusted for age, sex; Model **3**: adjusted for age, sex, current smoking, BMI, diabetes status, hypertension status, hypercholesterolemia status, history of cardiovascular disease, history of cancer, Vitamin K antagonists, Antiplatelet medication, Heparin use, Oral contraceptives

Interestingly, when cause specific mortality was considered, low AT levels were associated with lower risk of cardiovascular disease-related mortality after full adjustment (HR_Q1vsQ5_: 0.64, 95% CI:0.44–0.91, model 3; Table 2). On the contrary, low levels of AT were associated with high cancer-related mortality risk in crude analysis and after adjustment for age and sex (HR_Q1vsQ5_ 3.26, 95% CI: 2.34–4.55, model 1 and HR_Q1vsQ5_ 1.51, 95% CI:1.06–2.14, model 2, respectively; Table 2). However, further adjustment for other confounders, reduced the association (HR_Q1vsQ5_ 1.26, 95% CI:0.88–1.81, model 3; Table 2).

**Fig 3:**
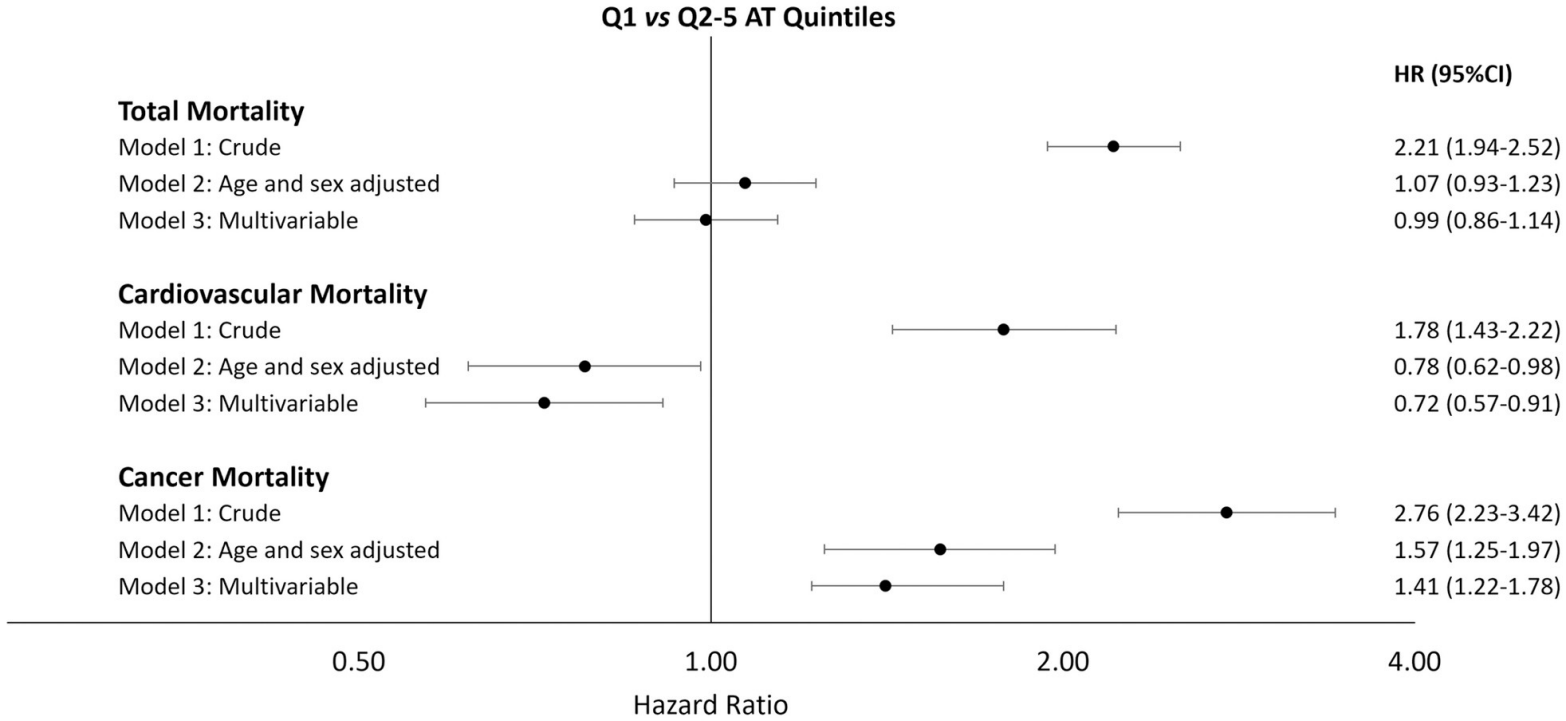
Hazard ratios (95% CI) for total, cardiovascular and cancer mortality comparing Q1 (AT <94.3%) vs Q2-5 (AT ≥ 94.3%), in the Moli-sani population (n = 19,676).

The association with the risk of death was particularly evident for the lowest quintile of AT (Q1), therefore hazard ratios were re-calculated using the group of individuals with AT ≥ 94.3% (Q2-5) as the reference group. In multivariable analysis, as illustrated in Fig 3 and S1 Table, AT levels below 94.3% were not associated with all-cause mortality (HR_Q1vsQ2-5_: 0.99, 95% CI: 0.86–1.14, model 3), but were associated with lower risk of cardiovascular disease-related mortality (HR_Q1vsQ2-5_: 0.72, 95% CI: 0.57–0.91, model 3) and with a higher risk of cancer-related mortality (HR_Q1vsQ2-5_: 1.41, 95% CI: 1.22–1.78, model 3). The result did not change after exclusion of subjects with AT level below 70% (data not shown).

Additionally, we investigated the association of AT levels with the occurrence of thrombotic events such as coronary heart disease (CHD) and stroke (S2 Table). During the follow-up period, 367 subjects suffered from a CHD event, and 62 subjects developed a stroke. Although low AT showed a significant association with the occurrence of CHD, this association was attributed to differences in age and sex between the quintiles of AT. No significant association was found between AT levels and the occurrence of stroke.

## Discussion

For the first time, the prospective relationship between AT levels and mortality was investigated in the general population. Interestingly, AT levels below 94.3% were significantly associated with a reduced risk of cardiovascular disease-related deaths after adjustment for age, sex, and several possible confounders. This was unexpected as published data showed that AT deficiency (below the normal range) was associated with venous thrombosis, although a clear relation with arterial thrombosis has never been demonstrated [10–13]. Several reports have observed an association between deficient AT levels and mortality in cancer patients [19–21] or severely ill patients [22]. Indeed, in contrast to CVD-related mortality, we did find that low AT levels were associated with cancer related mortality.

The relationship of AT and mortality has been studied in several patient populations, mostly with a case-control design and considering AT levels largely below the normal range of AT distribution, due to the severity of their disease. For example, in acute pancreatitis, abnormal AT levels were inversely associated with the risk of mortality [22]. The studies comparing the difference in AT between subjects that survived or died during a specific follow-up period usually did not correct for age and sex [19, 20]. In our study we also show that low AT levels were associated with higher cardiovascular mortality in the crude Cox regression model. However, since low AT levels were more common in men and in subjects at older age or with cardiovascular risk factors, correction for such possible confounders showed that subjects with low AT levels were actually protected against cardiovascular death. This finding is supported by the study of Azuhata et al. in sepsis patients, which reported reduced mortality in patients with AT levels between 70%-100% compared to patients with AT levels below 70% or above 100% [23]. Choi et al. showed that AT levels below 70% were associated with an increased risk of stroke and left atrial thrombus formation in patients with atrial fibrillation [24, 25]. Inherited AT deficiency was shown to be associated with an excess of mortality from venous thromboembolism [25], although another study on a large number of AT-deficient families did not find excess mortality for total or cardiovascular mortality [26]. The relation between AT levels and coronary artery disease is also questionable. Indeed, elevated AT levels compared with controls have been described in patients with acute myocardial infarction [27], explained as a reflection of a homeostatic response to plaque rupture and thrombosis. In contrast, Glader et al. found an inverse association between AT levels and risk of death in patients with coronary artery disease [28].

We observed for the first time, that subjects in the lowest quintile of AT distribution in a general population are protected from cardiovascular disease-related death. A possible explanation for this phenomenon could be that the overall combination of pro- and anticoagulant factor levels in the normal population are more favorable in subjects with low rather than high AT levels. In healthy subjects, a significant positive correlation between for example AT and fibrinogen has been reported, indicating that although AT levels are lower, this is compensated on an individual basis by a decrease in procoagulant factors such as fibrinogen [29]. Additionally, we found a significant correlation between AT levels and prothrombin, FV and FX levels in a subset of 120 healthy subjects (S2 Fig).

Moreover, in the case of acquired AT deficiency, which occurs for example in liver cirrhosis patients, the decrease in AT levels is paralleled by a reduction of pro-coagulant factors [30]. Interestingly, in the field of hemophilia, AT levels are decreased therapeutically to restore the hemostatic balance. In the phase 1 trial of the AT targeting drug fitusiran, healthy donors were infused with an miRNA to decrease plasma AT levels to approximately 80% of normal, and none of the subjects suffered from thrombosis-related complications [15].

In contrast to CVD mortality, we found a significant association between low normal range AT levels and cancer mortality. The relationship of AT levels and cancer mortality has been investigated in case-control studies and survival studies in diagnosed cancer patients with a relatively short follow-up period [19, 20]. Low AT levels have been associated with a higher mortality in patients with different types of cancer. In ovarian cancer, survivors are reported to have a 16% higher AT level compared to non-survivors [19, 20]. Sun et al showed a borderline significant lower survival rate for pancreatic cancer patients with AT levels below the median [31]. In patients with lymphoproliferative disorders, low AT was significantly associated with reduced survival [21]. In contrast, low AT levels are not associated with a lower survival rate in lung cancer patients [32, 33] Moreover, high plasma levels of the complex of thrombin with AT (T-AT) were found significantly associated with lower survival in cancer [34, 35]. Generally, T-AT formation occurs in parallel with reduction of AT due to consumption of AT. Thus, the association of high T-AT with higher cancer mortality corroborates our finding that low AT levels are associated with lower cancer survival.

AT is an established therapeutic target within hemostasis; such therapeutic efficacy is affected by the natural variation in AT plasma levels. For example, variation in AT levels can cause a variation in response to heparin treatment. Moreover, in bleeding disorders such as hemophilia A, AT is targeted to reduce the plasma level within the normal range values to achieve a better hemostatic balance. From previous case-control cohort studies it could be expected that low AT levels are a risk factor for mortality. Our results however show that lower normal range AT levels (below 94.3%) are associated with a reduced risk of CVD mortality. In cancer patients, the early detection of coagulation disorders such as low AT levels could allow tailoring of anticoagulant treatment and thereby preventing thromboembolism [36]. Treatment with AT concentrates during cancer therapy has been shown to decrease the number of thrombotic events and improve survival [37, 38]. Moreover, sufficient AT is pivotal to ensure the efficiency of heparin therapy, which is the anticoagulant of choice in cancer patients.

There are multiple clinical implications for the findings of our study. The novel finding of the association of low AT levels and a low risk of CVD death is of general clinical interest. Lower AT levels are intuitively consider to be unsafe in the light of the risk of thrombosis, as it is the most important naturally occurring anticoagulant. However, our results show that the slight lowering of AT levels might reduce the risk of CVD death rather than increase it. Moreover, coagulation and the pathogenesis of thrombosis is a complex mechanism that is not solely determined on AT. Nevertheless, our data suggest that even after correction for CVD-associated confounders such as a history of CVD, or hypertension, low AT are significantly associated with a reduced risk of CVD death. The causal relationship remains to be elucidated in more detailed studies, although the literature suggest that the positive association between AT and fibrinogen, FV, and FX might partially explain our findings. Our results regarding cancer mortality suggest that AT might be a good, early biomarker for cancer mortality. Interestingly, the burden of thrombosis risk is high in cancer patients, and the standard anticoagulant prescribed to cancer patients is heparin, although it has been proposed that direct oral anticoagulants might be beneficial in cancer patients [39]. Our findings indirectly suggest that cancer patients with low AT might benefit from other types of anticoagulants, as the death rate in subjects with low AT levels is higher compared to subjects with higher AT levels, and thrombosis is an important cause of death in cancer patients [39].

This study has several minor limitations. The studied end points, for example, only reflect part of the spectrum of causes of death, as we have chosen to study total mortality, cardiovascular disease-related mortality and cancer mortality. However, as cardiovascular diseases (34%) or cancer (18%)-related death contribute to more than half of the global annual mortality, they are important outcomes for mortality analysis. In addition to mortality analyses, we performed analyses to study the relationship of AT and stroke and myocardial infarction (MI). Although the associations found in the crude and mildly adjusted models were statistically significant, models corrected for CVD at baseline and anticoagulant usage were not, presumably due to the low number of strokes and MI events recorded during the follow-up. A third limitation to be considered is the long term storage of plasma samples. However, the samples were stored in liquid nitrogen, and we performed quality controls by measuring the most labile coagulation factors (V and FVIII) in a sub-cohort to confirm the good quality of the samples. A fourth limitation was the lack of information on the history of venous thromboembolism in the study. Lastly, AT levels were only determined in the samples obtained during the baseline blood collection. AT levels are know to decrease with age, and in the cancer population, heparin treatment can induce a mild AT deficiency due to its consumption in vivo. Therefore, it is likely that AT levels at the time of the CVD- or cancer-related death, were different from the baseline level. Nevertheless, in this manuscript we aim to study the role of AT as an early biomarker of CVD death and cancer death, which was achieved in the current analyses.

In conclusion, we show that low AT levels significantly reduce the risk of fatal cardiovascular events in the general population, regardless of age, sex and medication use. Moreover, we confirm that, in contrast to CVD survival, low AT levels are associated with lower cancer survival and we show for the first time that lower normal range AT levels in the general population, even before the development or diagnosis of cancer, are associated with an elevated risk of cancer death.

## Supporting information

S1 Fig Antithrombin distribution in whole sample in the Moli-sani population (n = 19,676). (PDF)

S2 Fig The correlation of antithrombin levels with procoagulant factor levels in 120 healthy subjects. (A) AT and prothrombin levels are significantly correlated (R = 0.281, p = 0.002). (B) AT and FV levels are significantly correlated (R = 0.219, p = 0.019). (C) AT and FX levels are significantly correlated (R = 0.233, p = 0.012).
(PDF)

S1 Table Hazard ratios (95% CI) for total, cardiovascular and cancer mortality comparing Q1 (AT <94.3%) vs Q2-5(AT ≥ 94.3%), in the Moli-sani population (n = 19,676). (PDF)

S2 Table Incidence and hazard ratios (95% CI) for coronary heart disease and stroke events according to quintiles of AT %, in the Moli-sani population. (PDF)

S1 Appendix Moli-sani study investigators. (PDF)
