# Supplementary figures and images for: Low antithrombin levels are associated with low risk of cardiovascular death but are a risk factor for cancer mortality
Source: PLoS One. 2022 Sep 19;17(9):e0271663. doi: 10.1371/journal.pone.0271663 (PMC9484666; doi:10.1371/journal.pone.0271663)

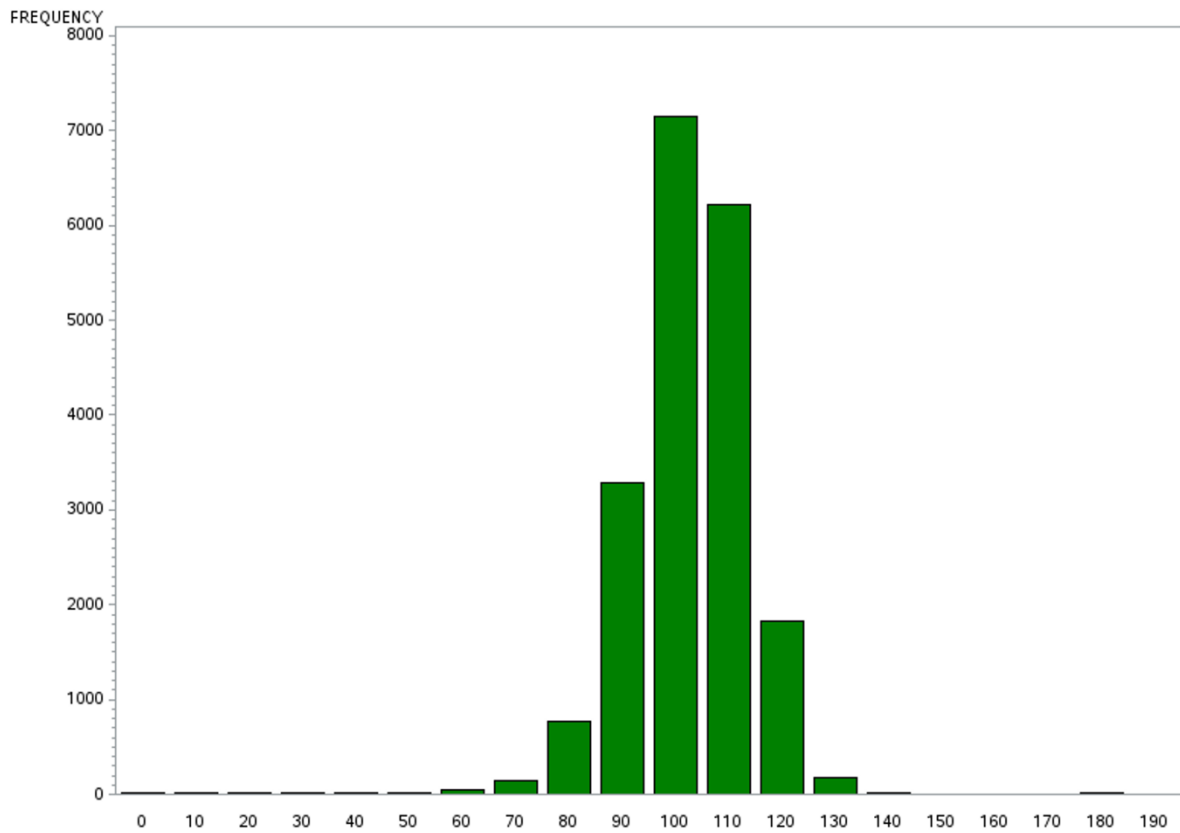

| AT      | Frequency | %     |
|---------|-----------|-------|
| < 71%   | 142       | 0.72  |
| 71-130% | 19,490    | 99.05 |
| >130%   | 44        | 0.22  |

**S1 Fig. Antithrombin distribution in whole sample in the Moli-sani population (n= 19,676).**

Supplement: S1 Fig — (PDF) [file pone.0271663.s001.pdf]
