# Supplementary material for: Low antithrombin levels are associated with low risk of cardiovascular death but are a risk factor for cancer mortality
Source: PLoS One. 2022 Sep 19;17(9):e0271663. doi: 10.1371/journal.pone.0271663 (PMC9484666; doi:10.1371/journal.pone.0271663)
Supplement: S2 Fig — (A) AT and prothrombin levels are significantly correlated (R = 0.281, p = 0.002). (B) AT and FV levels are significantly correlated (R = 0.219, p = 0.019). (C) AT and FX levels are significantly correlated (R = 0.233, p = 0.012). (PDF) [file pone.0271663.s002.pdf]

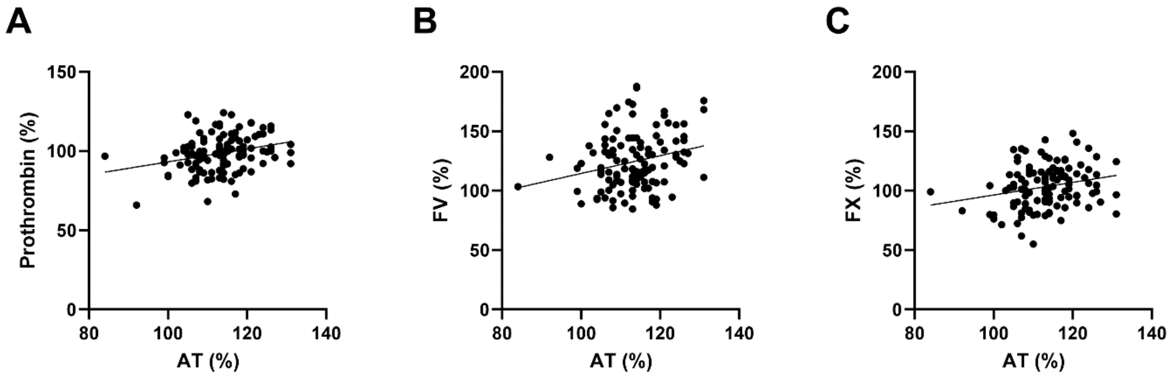

S2 Fig. The correlation of antithrombin levels with procoagulant factor levels in 120 healthy subjects. (A) AT and prothrombin levels are significantly correlated (R=0.281, p=0.002). (B) AT and FV levels are significantly correlated (R=0.219, p=0.019). (C) AT and FX levels are significantly correlated (R=0.233, p=0.012).
