# Supplementary material for: Low antithrombin levels are associated with low risk of cardiovascular death but are a risk factor for cancer mortality
Source: PLoS One. 2022 Sep 19;17(9):e0271663. doi: 10.1371/journal.pone.0271663 (PMC9484666; doi:10.1371/journal.pone.0271663)
Supplement: S1 Table — (PDF) [file pone.0271663.s003.pdf]

S1 Table. Hazard Ratios (95% CI) for Total, Cardiovascular and Cancer Mortality comparing Q1 (AT <94.3 %) vs Q2-5(AT ≥ 94.3 %), in the Moli-sani population (n= 19,676).

	**Q1**<br>**AT < 94.3 %**	**Q2-5**<br>**AT ≥ 94.3 %**	**P value**
**Total mortality**			
**model 1**	2.21 (1.94-2.52)	-1-	<.0001
**model 2**	1.07 (0.93-1.23)	-1-	0.35
**model 3**	0.99 (0.86-1.14)	-1-	0.84
**CVD Mortality**			
**model 1**	1.78 (1.43-2.22)	-1-	<.0001
**model 2**	0.78 (0.62-0.98)	-1-	0.036
**model 3**	0.72 (0.57-0.91)	-1-	0.0058
**Cancer Mortality**			
**model 1**	2.76 (2.23-3.42)	-1-	<.0001
**model 2**	1.57 (1.25-1.97)	-1-	<.0001
**model 3**	1.41 (1.22-1.78)	-1-	0.0033

Model **1**: crude; Model **2**: adjusted for age, sex; Model **3**: adjusted for age, sex, current smoking, BMI, diabetes status, hypertension status, hypercholesterolemia status, history of cardiovascular disease, history of cancer, Vitamin K antagonists, Antiplatelet medication, Heparin use, Oral contraceptives
