# Supplementary material for: Low antithrombin levels are associated with low risk of cardiovascular death but are a risk factor for cancer mortality
Source: PLoS One. 2022 Sep 19;17(9):e0271663. doi: 10.1371/journal.pone.0271663 (PMC9484666; doi:10.1371/journal.pone.0271663)
Supplement: S2 Table — (PDF) [file pone.0271663.s004.pdf]

**S2 Table. Incidence and Hazard Ratios (95% CI) for Coronary Heart Disease and Stroke Events according to quintiles of AT %, in the Moli-sani population.**

| | Quintiles | | | | | *p-value* |
|---|---|---|---|---|---|---|
| | **Q1** | **Q2** | **Q3** | **Q4** | **Q5** | |
| **Range** | **AT < 94.3%** | **94.3% ≤ AT < 100.5%** | **100.5% ≤ AT < 105.4%** | **105.4% ≤ AT < 111.1%** | **AT ≥ 111.1%** | |
| **CHD events** | | | | | | |
| **N** | 3936 | 3935 | 3937 | 3922 | 3934 | |
| **Person Years** | 9223.4 | 9223.4 | 9223.4 | 32694.3 | 9223.4 | |
| **N of events (rate)** | 116 (2.9) | 90 (2.3) | 66 (1.7) | 50 (1.3) | 45 (1.1) | |
| **model 1** | 2.74 (1.94-3.86) | 2.08 (1.45-2.97) | 1.50 (1.02-2.19) | 1.12 (0.75-1.68) | -1- | <.0001 |
| **model 2** | 1.05 (0.73-1.51) | 1.21 (0.84-1.74) | 1.12 (0.76-1.63) | 0.96 (0.64-1.43) | -1- | 0.680 |
| **Stroke** | | | | | | |
| **N** | 3936 | 3935 | 3937 | 3934 | 3934 | |
| **Person Years** | 9223.4 | 9223.4 | 9223.4 | 9223.4 | 9223.4 | |
| **N of events (rate)** | 15 (0.4) | 17 (0.4) | 11 (0.3) | 8 (0.2) | 11 (0.3) | |
| **model 1** | 1.45 (0.66-3.16) | 1.62 (0.76-3.45) | 1.02 (0.44-2.35) | 0. 74 (0.30-1.83) | -1- | 0.312 |
| **model 2** | 0.60 (0.26-1.40) | 0.99 (0.46-2.14) | 0.79 (0.34-1.84) | 0.63 (0.25-1.57) | -1- | 0.581 |

Model **1**: crude; Model **2**: adjusted for age, sex.
