# Supplementary material for: Low antithrombin levels are associated with low risk of cardiovascular death but are a risk factor for cancer mortality
Source: PLoS One. 2022 Sep 19;17(9):e0271663. doi: 10.1371/journal.pone.0271663 (PMC9484666; doi:10.1371/journal.pone.0271663)
Supplement: S1 Appendix — (PDF) [file pone.0271663.s005.pdf]

# Appendix S1: Moli-sani Study Investigators

The enrolment phase of the Moli-sani Study was conducted at the Research Laboratories of the Catholic University in Campobasso (Italy), the follow up of the Moli-sani cohort is being conducted at the Department of Epidemiology and Prevention of the IRCCS Neuromed, Pozzilli, Italy.

Steering Committee: Licia Iacoviello*°(Chairperson), Giovanni de Gaetano* and Maria Benedetta Donati*.

Scientific secretariat: Marialaura Bonaccio*, Americo Bonanni*, Chiara Cerletti*, Simona Costanzo*, Amalia De Curtis*, Augusto Di Castelnuovo§, Francesco Gianfagna°§, Mariarosaria Persichillo*, Teresa Di Prospero* (Secretary).

Safety and Ethical Committee: Jos Vermylen (Catholic Univesity, Leuven, Belgium) (Chairperson), Ignacio De Paula Carrasco (Accademia Pontificia Pro Vita, Roma, Italy), Antonio Spagnuolo (Catholic University, Roma, Italy).

External Event adjudicating Committee: Deodato Assanelli (Brescia, Italy), Vincenzo Centritto (Campobasso, Italy).

Baseline and Follow-up data management: Simona Costanzo* (Coordinator), Marco Olivieri (Associazione Cuore Sano, Campobasso, Italy), Teresa Panzera*.

Data Analysis: Augusto Di Castelnuovo§ (Coordinator), Marialaura Bonaccio*, Simona Costanzo*, Simona Esposito*, Alessandro Gialluisi*, Francesco Gianfagna°§, Emilia Ruggiero*.

Biobank and biochemical laboratory: Amalia De Curtis* (Coordinator), Sara Magnacca§.

Genetic laboratory: Benedetta Izzi* (Coordinator), Annalisa Marotta*, Fabrizia Noro*, Roberta Parisi*, Alfonsina Tirozzi*.

Recruitment staff: Mariarosaria Persichillo* (Coordinator), Francesca Bracone*, Francesca De Lucia (Associazione Cuore Sano, Campobasso, Italy), Cristiana Mignogna°, Teresa Panzera*, Livia Rago*.

Communication and Press Office: Americo Bonanni*.

Regional Health Institutions: Direzione Generale per la Salute - Regione Molise; Azienda Sanitaria Regionale del Molise (ASReM, Italy); Molise Dati Spa (Campobasso, Italy); Offices of vital statistics of the Molise region.

Hospitals: Presidi Ospedalieri ASReM: Ospedale A. Cardarelli – Campobasso, Ospedale F. Veneziale – Isernia, Ospedale San Timoteo - Termoli (CB), Ospedale Ss. Rosario - Venafro (IS), Ospedale Vietri – Larino

(CB), Ospedale San Francesco Caracciolo - Agnone (IS); Casa di Cura Villa Maria - Campobasso; Ospedale Gemelli Molise - Campobasso; IRCCS Neuromed - Pozzilli (IS).

*Department of Epidemiology and Prevention, IRCCS Neuromed, Pozzilli, Italy
°Department of Medicine and Surgery, University of Insubria, Varese, Italy
§Mediterranea Cardiocentro, Napoli, Italy

*Baseline Recruitment staff is available at https://www.moli-sani.org/?page_id=173*
